# Nutrient enrichment shifts mangrove height distribution: Implications for coastal woody encroachment

**DOI:** 10.1371/journal.pone.0193617

**Published:** 2018-03-01

**Authors:** Carolyn A. Weaver, Anna R. Armitage

**Affiliations:** 1 Department of Ecosystem Science and Management, Texas A&M University, College Station, Texas, United States of America; 2 Department of Marine Biology, Texas A&M University at Galveston, Galveston, Texas, United States of America; North University of China, CHINA

## Abstract

Global changes, such as increased temperatures and elevated CO_2_, are driving shifts in plant species distribution and dominance, like woody plant encroachment into grasslands. Local factors within these ecotones can influence the rate of regime shifts. Woody encroachment is occurring worldwide, though there has been limited research within coastal systems, where mangrove (woody shrub/tree) stands are expanding into salt marsh areas. Because coastal systems are exposed to various degrees of nutrient input, we investigated how nutrient enrichment may locally impact mangrove stand expansion and salt marsh displacement over time. We fertilized naturally co-occurring *Avicennia germinans* (black mangrove) and *Spartina alterniflora* (smooth cordgrass) stands in Port Aransas, TX, an area experiencing mangrove encroachment within the Northern Gulf of Mexico mangrove-marsh ecotone. After four growing seasons (2010–2013) of continuous fertilization, *Avicennia* was more positively influenced by nutrient enrichment than *Spartina*. Most notably, fertilized plots had a higher density of taller (> 0.5 m) mangroves and mangrove maximum height was 46% taller than in control plots. Fertilization may promote an increase in mangrove stand expansion within the mangrove-marsh ecotone by shifting *Avicennia* height distribution. *Avicennia* individuals, which reach certain species-specific height thresholds, have reduced negative neighbor effects and have higher resilience to freezing temperatures, which may increase mangrove competitive advantage over marsh grass. Therefore, we propose that nutrient enrichment, which augments mangrove height, could act locally as a positive feedback to mangrove encroachment, by reducing mangrove growth suppression factors, thereby accelerating the rates of increased mangrove coverage and subsequent marsh displacement. Areas within the mangrove-marsh ecotone with high anthropogenic nutrient input may be at increased risk of a regime shift from grass to woody dominated ecosystems.

## Introduction

Global changes are driving shifts in plant species coverage, phenology, and distribution within multiple biomes around the world [1]. Species within ecotones, defined as intermediate areas between different vegetation types, are particularly sensitive to global changes [2]. A shift in dominant vegetation type could dramatically alter associated ecosystem services [3]. Therefore, it is imperative to understand how global changes may influence dominant plant species presence within an ecotone.

Oscillations in dominant vegetation types in the ecotones between terrestrial grasslands and shrublands can be mediated by many factors and are thought to be heavily influenced by global changes [4]. Over the past two centuries, woody vegetation has expanded globally in biomass and coverage, often encroaching into grasslands [5, 6]. Woody encroachment is influenced by global changes such as increases in temperatures or elevated CO_2_ [7, 8]. Other, generally local, factors such as intensified grazing practices and reduced fire occurrence, can further influence this vegetation shift [7, 9].

Although most literature has focused on terrestrial woody encroachment, this phenomenon is also occurring along the coast within the mangrove-marsh ecotone [6]. Mangroves are woody halophytes common in tropical coastal systems. Mangrove distribution is influenced by a variety of environmental parameters [10], but temperature and precipitation are the environmental factors most closely linked to mangrove global distribution and latitudinal range limits [11]. Over the last 50 years, mangrove stands have increased in these ecotonal regions and have expanded poleward on five continents, often encroaching into salt marshes dominated by herbaceous halophytes [12, 13].

Mangrove encroachment within mangrove-marsh ecotones has often been attributed to global changes such as sea level rise [13] and reduction in the frequency, duration, and severity of freezing events [14, 15]. Coastal woody encroachment may be similar to terrestrial woody expansion in that other local factors may further influence this habitat shift. Coastal systems such as mangrove and marsh stands are highly susceptible to anthropogenic nutrient enrichment from runoff and wastewater discharge [16–18]. Fertilization generally increases plant growth and productivity in monotypic stands of either mangrove (e.g., [19, 20]) or marsh (e.g., [21, 22]) vegetation. However, the effects of *in situ* nutrient enrichment within mixed, mature stands of mangrove and marsh vegetation have not been documented; therefore, it is unclear how nutrient enrichment may influence the dynamics of mangrove encroachment. Because marsh plants can suppress mangrove growth and survival [23–25] and marsh grasses may be a better competitor for nutrient resources [26, 27], marsh vegetation growth responses to nutrient enrichment may be greater than those of mangroves. Along the expanding edge of a mangrove stand, where mangrove and marsh plants co-occur, mangroves are smaller and may experience negative interactions with neighboring salt marsh plants. In nutrient enriched conditions, this growth suppression may be further augmented by accelerating marsh plant growth and subsequently reducing mangrove growth [23, 26].Therefore, nutrient enrichment may slow the encroachment of mangroves by maintaining the dominance of salt marsh species.

**Fig 1:**
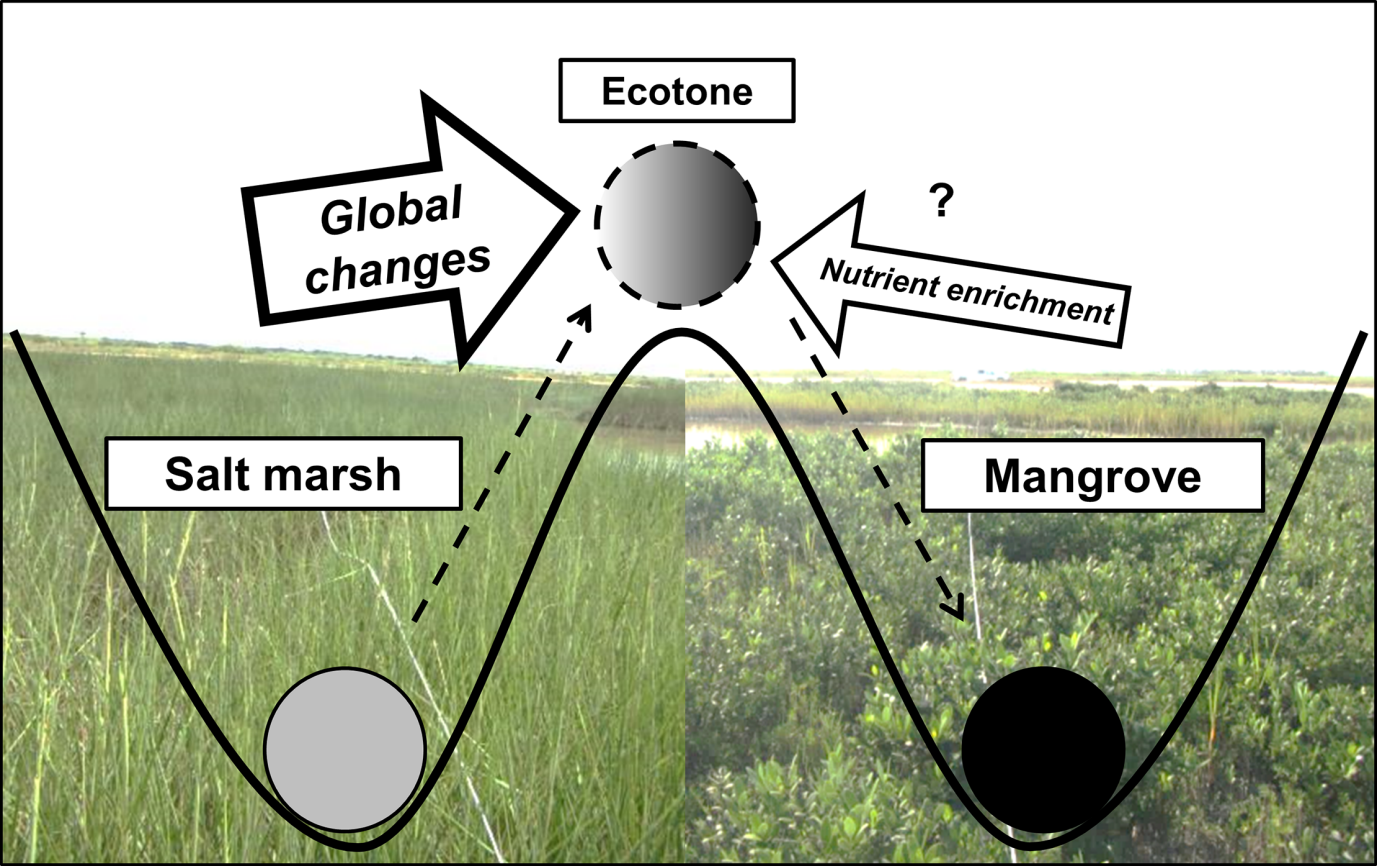
A conceptual ball-in-cup diagram hypothesizing the direction nutrient enrichment may drive the mangrove-marsh ecotone. Global changes (e.g., reductions in freezing events, sea level rise, and higher atmospheric CO_2_) are often evoked as the main driver in increased mangrove coverage. Nutrient enrichment, on a local scale, may augment marsh growth and reduce mangrove growth, subsequently contributing to slower mangrove encroachment.

Mangrove encroachment into salt marshes is accelerated by large-scale drivers like sea level rise and decreased freezing events, but local environmental factors can further influence this regime shift. To investigate if nutrient enrichment has a positive or negative effect on mangrove encroachment, we fertilized naturally occurring mixed stands of mangrove (*Avicennia germinans*–black mangrove) and marsh (primarily *Spartina alterniflora*–smooth cordgrass) vegetation on the Texas (USA) coast in the Northern Gulf of Mexico over four growing seasons (2010–2013). Plots were placed in an area where mangrove stands are actively increasing and replacing salt marsh [12] to investigate how nutrient addition may influence mangrove stand expansion and marsh displacement over time. Based on previous studies [23, 26], we expected that nutrient enrichment would augment marsh growth and conversely inhibit mangrove growth, particularly in smaller individuals. Therefore, we hypothesized that fertilization would decrease the magnitude of mangrove stand expansion, as represented by lower mangrove density and height, and maintain marsh dominance (Fig 1).

## Materials and methods

### Site description and experimental design

*Avicennia germinans* (black mangrove, hereafter *Avicennia*) is the most frequent mangrove species found in the Northern Gulf of Mexico [28]. It has been distributed across this region at least since 1853, typically occurring in small, discontinuous patches within larger expanses of marsh vegetation [13, 29]. Although *Avicennia* has a higher tolerance to cold temperatures than other mangrove species, this species is still susceptible to diebacks following severe freezing events [30]. Therefore, *Avicennia* in this region are often interspersed with marsh forb and graminoid species, particularly *Spartina alterniflora* (smooth cordgrass, hereafter *Spartina*) [31].

Port Aransas, TX, USA is one of the locations where persistent populations of *Avicennia* on the Texas coast have been documented since the 1930s [28, 29]. A massive mangrove dieback occurred in this region following several freezing events in the early 1980s [28, 29, 31], but since that time, hard freeze events have not been of sufficient severity (days with minimum temperature < -4°C [15]) to cause substantial dieback, and local mangrove stands have increased in areal cover [12, 31, 32]. In the last twenty years, mangrove coverage has surpassed the reported accounts in 1979, and most of this increase has been in areas previously dominated by salt marsh species, such as *Spartina* [12, 13, 31]. Because Port Aransas is within the Northern Gulf of Mexico mangrove-marsh ecotone and is actively experiencing mangrove encroachment, it was an ideal location to study how nutrient enrichment may influence this vegetation shift.

**Fig 2:**
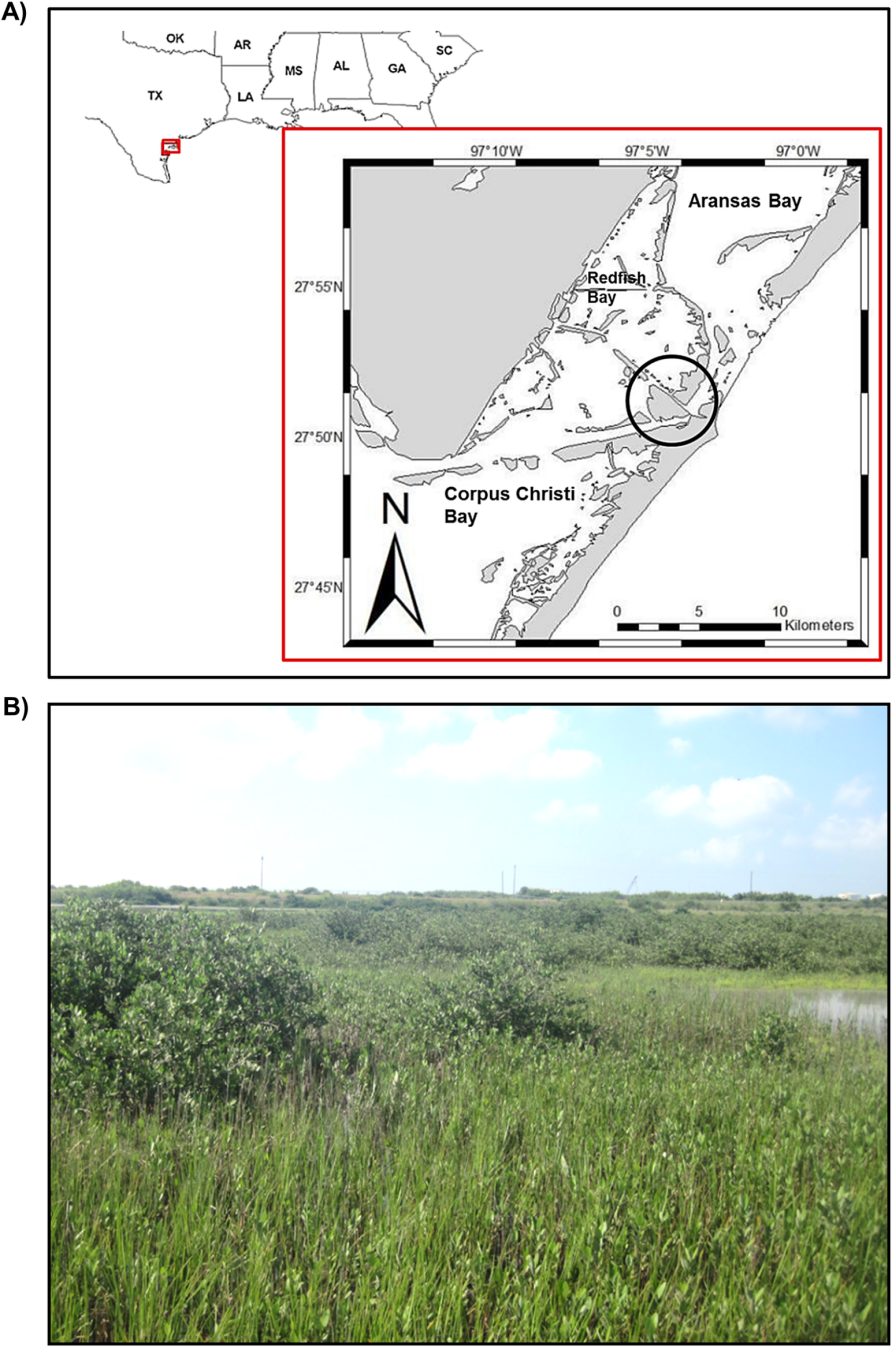
Study site location. (A) Plots were located in Port Aransas, TX, USA (B) in co-occurring *Avicennia germinans* (black mangrove) and *Spartina alterniflora* (smooth cordgrass) stands.

In the spring of 2010, plots were demarcated in Port Aransas (27.9°N, 97.1°W, Fig 2A) along the low marsh elevation contour, such that all plots experienced similar tidal inundation. During the course of the study (2010–2013), the average daily temperature was 21.80 °C (max: 31.40 °C; min: -0.96 °C), with only three days in February 2011 below freezing (these data were collected and made freely available by NOAA/NDBC). Other abiotic data and site descriptions from the low marsh zone can be obtained from other studies within the same region [25, 33, 34]. Plots were located on public land and did not involve endangered or protected species.

Plots were demarcated where *Avicennia* was interspersed with characteristic low elevation marsh vegetation (Fig 2B), mainly *Spartina* [25]. The study period began in spring 2010 at the beginning of the *Spartina* (a perennial grass) annual growing season [35]. Plots were placed along the expanding edge of the mangrove stand in order to measure species interactions where *Avicennia* was encroaching into *Spartina*. At the time of plot deployment, mangroves were mostly (> 95%) less than 50 cm in height but some small shrubs were present; no individuals exceeded 150 cm. Succulent marsh species, primarily *Batis maritima* (saltwort) and *Salicornia depressa* (Virginia glasswort), were also present in and around the plots.

Plots were placed within the low marsh along a similar tidal elevation in a split block design where each of the eleven blocks (no closer than 4 m) contained two 4 m^2^ plots, one of each nutrient treatment type: control and fertilized. A randomized block design was used to account for landscape heterogeneity. Prior to treatment application, there were no significant differences between plots, based on species densities using a two-way mixed permutational analysis of variance (permANOVA; treatment x block). A slow-release fertilizer (Osmocote® Outdoor & Indoor Smart-Release® Plant Food NPK 19-6-12) was applied by broadcasting and gently massaging pellets into the sediment surface. Fertilizer was re-applied every two to three months; application amounts generated loading rates of 0.342 g N m^-2^ day^-2^ and 0.108 g P m^-2^ day^-1^. The fertilization technique and loading rates were selected based on previous enrichment experiments in Northern Gulf of Mexico salt marshes (e.g., [36]).

### Sample collection and analysis

Plots were sampled at peak plant production prior to *Spartina* senescence [35] each year from 2010 through 2013 (September–October). Total density of each species present was quantified for the entire plot (2 m x 2 m) or within representative subplots (30 cm x 30 cm); trunk and stem densities were standardized to number per square meter. *Avicennia* densities were recorded in each of three height classes: < 0.5 m, 0.5 m—1.0 m, and > 1.0 m (herein, sub-shrub, shrub, and tall shrub, respectively). Seedlings (as indicated by the presence of cotyledons) were minimally observed and were included with the smallest height class, sub-shrub (mangroves < 0.5 m). The maximum height of the tallest *Avicennia* and *Spartina* individual within each plot was measured. Green leaves (n = 20) were collected from representative *Avicennia* (all height classes) and *Spartina* throughout each plot for nutrient content analyses as a proxy for a nutrient treatment response. In the laboratory, leaves were rinsed to remove salt and adhered sediments and dried to constant mass in an oven at 60 °C. Entire samples were ground and homogenized with a Thomas Wiley® Mini-Mill. Total carbon (C) and nitrogen (N) content were quantified using a Costech ECS 4010 Elemental Analyzer; analytical variability ranged 2–5%, as determined by running National Institute of Standards and Technology standard reference material (SRM 1941-b). Total phosphorus (P) content was determined via a dry-oxidation, acid hydrolysis extraction followed by a colorimetric analysis on a Shimadzu UV-1800 Spectrophotometer [37].

### Data analyses

Individual responses to nutrient enrichment for each sampling event (i.e., density, height, and leaf nutrient content) were determined with separate three-way permutational analysis of variance models (PermANOVA), which were employed for data analysis because they are robust but do not require assumptions of data normality [38, 39]. In all permANOVA, treatment (control and fertilized) and year (2010–2013) were fixed factors and block (11 levels) was treated as a random factor. The three-way interaction term (treatment x year x block) was excluded from the model because there was no replication within blocks, typical of randomized block experimental designs. Significance for analyses was determined using permutation p values, which were obtained from 9999 unique permutations of the data. All data were analyzed using PERMANOVA+ version 1.0.5 in PRIMER 6 version 6.1.15 (PRIMER-E Ltd., Plymouth Marine Laboratory, UK) [39].

Total *Avicennia* and *Spartina* densities, as well as *Avicennia* height classes, were analyzed separately. Density data were fourth root transformed and Bray Curtis resemblance was used. To account for the high number of zeros within the *Avicennia* height class (sub-shrub, shrub, and tall shrub) and *Spartina* density data, a dummy variable was added to each resemblance matrix. Pairwise tests were used to identify significant differences between nutrient treatments and among sampling years.

Individual analyses for maximum height and nutrient content parameters (total % C, % N, % P, C:N, C:P, and N:P) for each species were conducted. Data were square root transformed and a Euclidean distance based resemblance matrix was used. Some *Avicennia* leaves collected in 2013 were contaminated in the laboratory and therefore nutrient data for the 2013 sampling event consisted of only six of the eleven blocks. In some plots, *Spartina* was not present (particularly in the final sampling event), and therefore those plots were excluded from the height and nutrient analyses.

## Results

**Fig 3:**
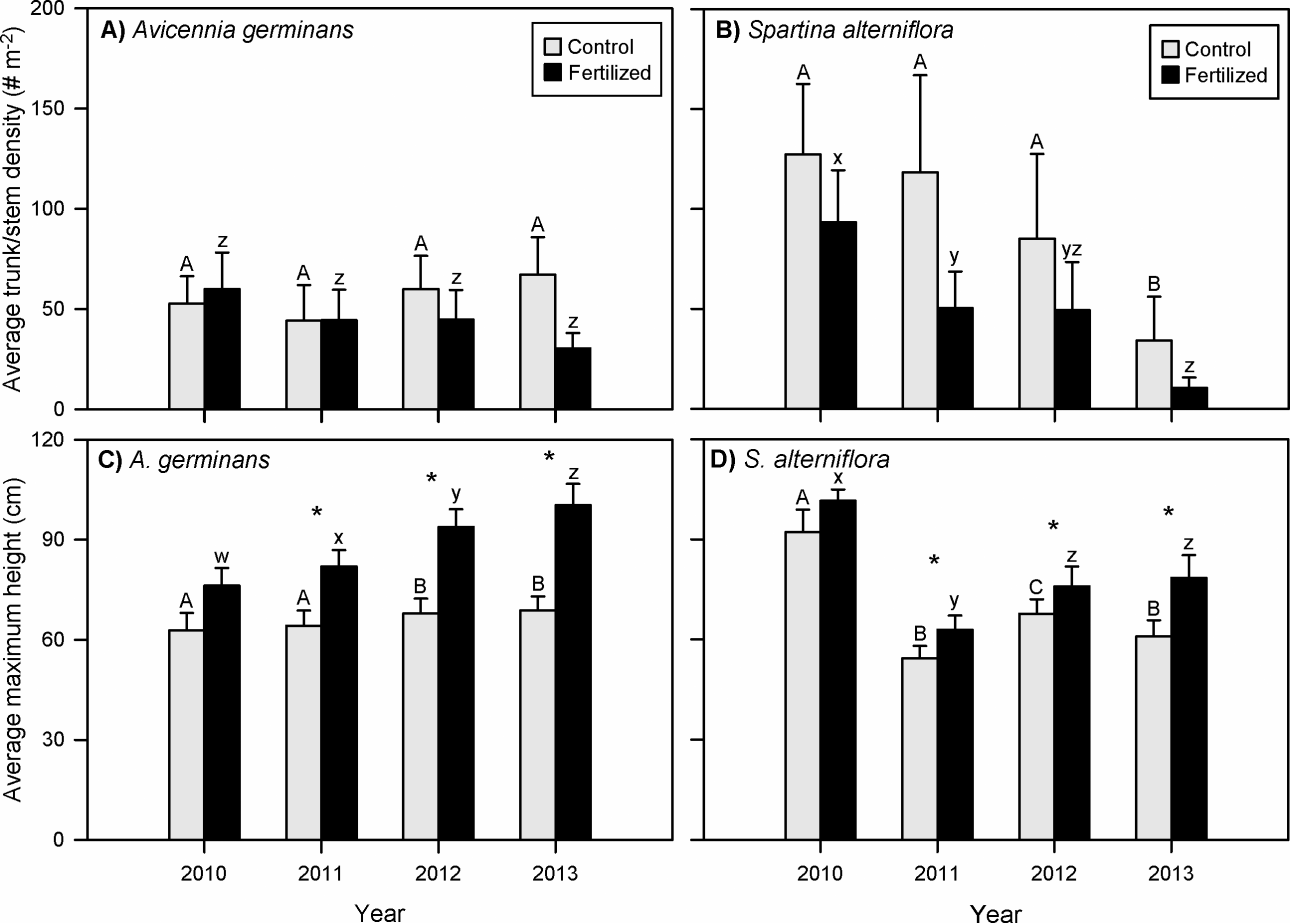
*Avicennia germinans* (black mangrove) and *Spartina alterniflora* (smooth cordgrass) density and height values. (A,C) *Avicennia* and (B,D) *Spartina* (smooth cordgrass) total trunk/stem densities per square meter (# m^-2^) and maximum heights (cm) from control (gray) and fertilized (black) treatment plots for each sampling year (2010–2013). Data are mean values ± standard error; n = 11. Upper case letters indicate temporal trends within control plots; lower case letters indicate temporal trends within fertilized plots. Different letters indicate significance at perm p < 0.05 within control or fertilized treatments; * indicates significance at perm p < 0.05 between treatments per year. (See Tables 1–3 for statistical analyses).

**Fig 4:**
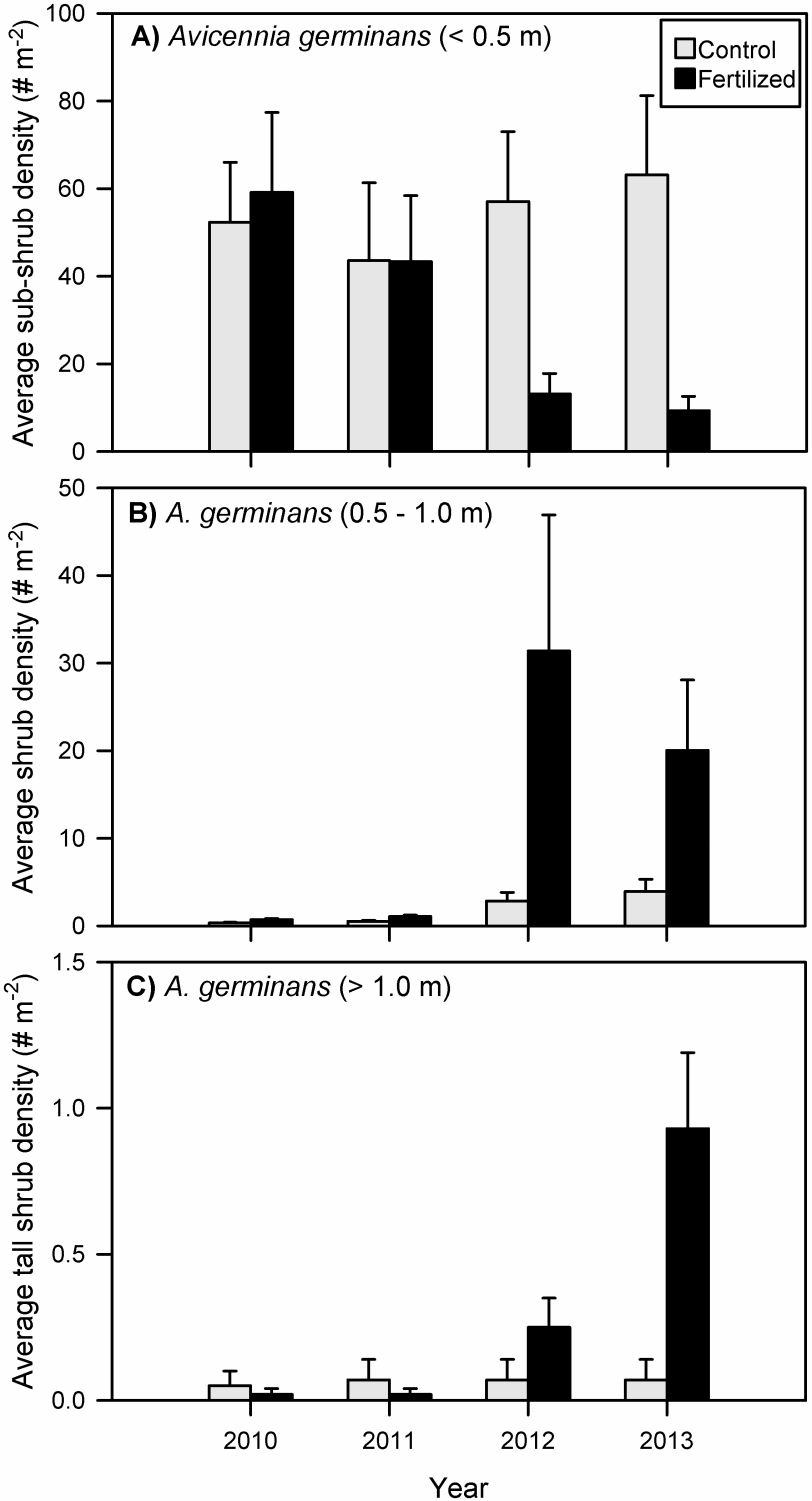
*Avicennia germinans* (black mangrove) height class densities. Black mangrove density per square meter (# m^-2^) values in control (gray) and fertilized (black) treatment plots categorized as (A) sub-shrub = *Avicennia* < 0.5 m, (B) shrub = *Avicennia* 0.5–1.0 m, and (C) tall shrub = *Avicennia* > 1.0 m within each sampling year (2010–2013). Data are mean values ± standard error; n = 11. Upper case letters indicate temporal trends within control plots; lower case letters indicate temporal trends within fertilized plots. Different letters indicate significance at perm p < 0.05 within control or fertilized treatments; * indicates significance at perm p < 0.05 between treatments per year. (See Tables 1–3 for statistical analyses).

Over the four growing seasons of the enrichment experiment, total *Avicennia* density did not change between fertilization treatments or over time (Fig 3A, Tables 1–3). When *Avicennia* plants were divided into height classes (sub-shrub, shrub, and tall shrub), treatment and temporal trends were evident. *Avicennia* sub-shrub and shrub densities were significantly different between treatments (Table 1). Fertilization shifted mangroves to taller height classes, as there were fewer sub-shrubs and more individuals in taller size classes in fertilized plots; this difference was particularly pronounced by the end of the third growing season in 2012 (Fig 4, Tables 1–3). Mangrove sub-shrub densities within control plots were similar across all four growing seasons, but significantly decreased over time within fertilized plots (Fig 4A). Shrub density increased over time in both treatments, but was ten times higher in fertilized than control plots in 2012 and 2013 (Fig 4B). Tall shrub density in control plots was constant over time, but significantly increased in fertilized plots throughout the course of the experiment (Fig 4C). *Spartina* density was not significantly different between treatments but significantly decreased over time in both treatment types; this temporal trend was more pronounced in fertilized plots (Fig 3B, Tables 1–3).

**Table 1:** PermANOVA results determining treatment and sampling year differences for density and maximum height values.

	*Avicennia germinans* (black mangrove)										*Spartina alterniflora* (smooth cordgrass)			
	Total density^a^		Sub-shrub density^b^		Shrub density^c^		Tall shrub density^d^		Maximum height		Total density		Maximum height	
	Pseudo F	Perm p	Pseudo F	Perm p	Pseudo F	Perm p	Pseudo F	Perm p	Pseudo F	Perm p	Pseudo F	Perm p	Pseudo F	Perm p
Treatment	0.47	0.53	7.23	0.02*	23.203	< 0.01*	0.12	0.83	15.92	< 0.01*	0.12	0.83	23.203	< 0.01*
Year	1.38	0.26	1.12	0.34	42.26	< 0.01*	10.97	< 0.01*	22.70	< 0.01*	10.97	< 0.01*	42.26	< 0.01*
Block	4.38	< 0.01*	2.46	0.02*	14.39	< 0.01*	10.64	< 0.01*	43.88	< 0.01*	10.64	< 0.01*	14.39	< 0.01*
Treatment x year	1.11	0.36	4.43	< 0.01*	2.29	0.11	0.30	0.93	8.31	< 0.01*	0.30	0.93	2.29	0.11
Year x block	1.41	0.16	0.97	0.54	1.77	0.11	1.29	0.18	1.39	1.19	1.29	0.18	1.77	0.11
Treatment x block	2.57	0.02*	1.58	0.14	2.00	0.10	9.23	< 0.01*	20.55	< 0.01*	9.23	< 0.01*	2.00	0.10

Results are from separate permANOVA to determine differences in *Avicennia germinans* (black mangrove; left portion) and *Spartina alterniflora* (smooth cordgrass; right portion) density per square meter (# m^-2^) and maximum height (cm) between treatment (control and fertilized) plots and sampling years (2010–2013). A three-way mixed permANOVA model was utilized: treatment (2 levels) x year (4 levels) x block (11 levels). Perm p values obtained from 9999 unique permutations of the data.

* Indicates significance at perm p < 0.05

^a^ *Avicennia* of all height classes

^b^ *Avicennia* < 0.5 m

^c^ *Avicennia* 0.5–1.0 m

^d^ *Avicennia* > 1.0 m.

**Table 2:** PermANOVA pairwise results comparing density and maximum height values between treatments.

***Avicennia germinans* (black mangrove)**								
**Year**	**2010**		**2011**		**2012**		**2013**	
	t	Perm p	t	Perm p	t	Perm p	t	Perm p
**Total density**^**a**^	0.31	0.86	0.55	0.60	1.35	0.20	1.97	0.07
**Sub-shrub density**^**b**^	0.35	0.78	0.40	0.71	2.74	0.02*	3.29	< 0.01*
**Shrub density**^**c**^	2.16	0.03*	3.57	< 0.01*	1.67	0.11	2.16	0.05
**Tall shrub density**^**d**^	0.09	0.77	0.14	0.77	1.71	0.12	4.09	< 0.01*
**Max height**	2.13	0.06	3.14	0.01*	4.38	< 0.01*	5.82	< 0.01*
***Spartina alterniflora* (smooth cordgrass)**								
**Year**	**2010**		**2011**		**2012**		**2013**	
	t	Perm p	t	Perm p	t	Perm p	t	Perm p
**Total density**	0.22	0.89	0.74	0.55	0.35	0.86	0.21	0.93
**Max height**	1.62	0.13	2.92	0.02*	2.87	0.03*	4.82	< 0.01*

Results are from separate pairwise permANOVA to determine treatment (control and fertilized) differences in *Avicennia germinans* (black mangrove; top portion) and *Spartina alterniflora* (smooth cordgrass; bottom portion) density per square meter (# m^-2^) and maximum height (cm) within each sampling year (2010–2013). A three-way mixed permANOVA model was utilized: treatment (2 levels) x year (4 levels) x block (11 levels). Significance was determined for treatment within each sampling year using a pairwise test (treatment x year). Perm p values obtained from 9999 unique permutations of the data.

* Indicates significance at perm p < 0.05

^a^ *Avicennia* of all height classes

^b^ *Avicennia* < 0.5 m

^c^ *Avicennia* 0.5–1.0 m

^d^ *Avicennia* > 1.0 m

**Table 3:** PermANOVA pairwise results comparing density and maximum height values between sampling years.

**Control plots**												
**Year**	**2010 x 2011**		**2010 x 2012**		**2010 x 2013**		**2011 x 2012**		**2011 x 2013**		**2012 x 2013**	
	t	Perm p	t	Perm p	t	Perm p	t	Perm p	t	Perm p	t	Perm p
**AG total density**^**a**^	0.81	0.43	0.83	0.45	0.86	0.44	1.99	0.07	2.11	0.06	0.45	0.67
**AG sub-shrub density**^**b**^	0.79	0.44	0.79	0.45	0.83	0.43	1.94	0.08	2.07	0.07	0.38	0.72
**AG shrub density**^**c**^	1.47	0.19	4.60	< 0.01*	4.48	< 0.01*	4.51	< 0.01*	4.39	< 0.01*	0.90	0.42
**AG tall shrub density**^**d**^	1.00	0.52	1.00	0.52	1.00	0.52	- - -	- - -	- - -	- - -	- - -	- - -
**AG max height**	1.48	0.18	2.39	0.03*	2.69	0.02*	2.59	0.03*	2.69	0.01*	0.90	0.39
**SA total density**	1.65	0.27	1.88	0.06	2.95	0.01*	1.65	0.08	3.11	< 0.01*	2.55	< 0.01*
**SA max height**	6.53	< 0.01*	5.29	< 0.01*	6.70	< 0.01*	3.70	< 0.01*	1.59	0.16	2.58	0.04*
**Fertilized plots**												
**Year**	**2010 x 2011**		**2010 x 2012**		**2010 x 2013**		**2011 x 2012**		**2011 x 2013**		**2012 x 2013**	
	t	Perm p	t	Perm p	t	Perm p	t	Perm p	t	Perm p	t	Perm p
**AG total density**^**a**^	0.37	0.76	0.59	0.59	0.83	0.44	0.62	0.59	0.99	0.39	0.66	0.59
**AG sub-shrub density**^**b**^	0.34	0.77	1.54	0.15	2.10	0.06	1.61	0.13	2.45	0.03*	1.46	0.18
**AG shrub density**^**c**^	4.47	< 0.01*	3.46	< 0.01*	4.40	< 0.01*	3.17	0.01*	3.93	< 0.01*	1.03	0.42
**AG tall shrub density**^**d**^	- - -	- - -	2.38	0.04*	4.15	< 0.01*	2.38	0.04*	4.15	< 0.01*	2.47	0.03*
**AG max height**	5.30	< 0.01*	4.52	< 0.01*	5.21	< 0.01*	3.57	< 0.01*	4.58	< 0.01*	2.54	0.03*
**SA total density**	2.28	0.04*	2.88	< 0.01*	4.07	< 0.01*	0.83	0.51	2.26	0.02*	1.46	0.13
**SA max height**	8.42	< 0.01*	4.19	< 0.01*	3.77	< 0.01*	2.69	0.03*	3.55	< 0.01*	0.69	0.51

Results are from separate pairwise permANOVA to determine differences in *Avicennia germinans* (black mangrove) and *Spartina alterniflora* (smooth cordgrass) density per square meter (# m^-2^) and maximum height (cm) values between sampling years (2010–2013) for control (top portion) and fertilized (bottom portion) plots. A three-way mixed permANOVA model was utilized: treatment (2 levels) x year (4 levels) x block (11 levels). Significance comparing sampling years was determined for each treatment type using a pairwise test (year x treatment). Perm p values obtained from 9999 unique permutations of the data.

AG = *Avicennia*

SA = *Spartina*

* Indicates significance at perm p < 0.05

^a^ *Avicennia* of all height classes

^b^ *Avicennia* < 0.5 m

^c^ *Avicennia* 0.5–1.0 m

^d^ *Avicennia* > 1.0 m

^- - -^ indicates “t” could not be calculated because a zero was present in the denominator (numbers were the same between years) and therefore a perm p was not assigned.

*Avicennia* maximum height in fertilized plots was significantly higher than the control in all years except the first sampling event (2010; Fig 3C, Tables 1–3). A temporal trend was evident in fertilized plots as maximum height of fertilized *Avicennia* significantly increased each year of the experiment (Fig 3C, Table 3). In control plots, *Avicennia* maximum height also increased over time, but by a much smaller margin than the fertilized counterparts; *Avicennia* maximum height significantly increased only between 2011 and 2012 (Fig 3C, Table 3). Maximum height was the only measured *Spartina* parameter that significantly differed between nutrient treatments. Fertilized *Spartina* was significantly taller than in control plots in all years following the first sampling event (Fig 3D, Tables 1–3). In both control and fertilized plots, *Spartina* was significantly taller in the first year (2010) than the subsequent sampling years (Fig 3D, Table 3).

*Avicennia* leaf nutrient content metrics, particularly measures of nitrogen content, significantly varied between nutrient treatments, whereas *Spartina* leaf nutrient contents did not (S1 Table). *Avicennia* had higher total % C in fertilized leaves in the first three years (2010–2012) and total leaf % N, C:N and N:P were significantly different between treatments in the second (2011) and third (2012) years (S2 and S3 Tables). Only *Avicennia* total leaf N:P was significantly higher in fertilized plots in the fourth growing season (2013), although total % N was near significant (perm p < 0.056). Fertilization did not significantly change *Spartina* leaf nutrient contents in any of the sampling years (S2 and S3 Tables).

## Discussion

### Species responses to nutrient addition

In order to assess how nutrient enrichment may affect mangrove encroachment within the mangrove-marsh ecotone, we fertilized naturally co-occurring *Avicennia* and *Spartina* stands for four growing seasons. *Avicennia* had more pronounced growth responses in fertilized plots than *Spartina*, most notably nutrient enrichment altered *Avicennia* size distribution and maximum height. Our findings were not what we anticipated; we hypothesized, based on previous mangrove and marsh fertilization studies, that the added nutrients would augment marsh growth and subsequently suppress mangrove growth. However, previous work focused on mangrove seedlings, documented nutrient responses over a smaller time scale, and/or were conducted in mesocosms [23, 26].

We hypothesized that nutrient enrichment would slow mangrove encroachment, which would be represented by lower *Avicennia* density within control plots compared to fertilized plots. We found though, that total *Avicennia* density was not significantly different between treatments nor was there a significant change in total density over the four growing seasons of the experiment (2010–2013). However, densities of the three mangrove size classes (sub-shrub, shrub, and tall shrub) did have a treatment response. Opposite of our hypothesis, the two taller mangrove size classes (shrub and tall shrub) increased over time in fertilized plots and were substantially higher than those in the control plots by the third growing season. In contrast, *Avicennia* sub-shrub density was constant over time within control plots but decreased in density in the fertilized plots over the four growing seasons. This decrease suggests that more individuals grew into the next height class in response to fertilization. The reduced number of smaller mangroves in fertilized plots may also indicate that there were few new mangrove recruits.

We anticipated that *Spartina* density would increase over time in fertilized plots. Contrary to our expectations, *Spartina* density was not significantly different between control and fertilized plots. Further, *Spartina* density declined over time in all treatments, likely driven by external abiotic factors (e.g., drought condition in 2011 that altered precipitation and temperature patterns [40]) that were beyond the scope of our study. Nutrient enrichment appeared to accelerate the rate of decrease, possibly due to the taller mangrove canopy that developed in fertilized plots.

Height is often a measurement used to detect a fertilizer-induced growth response. In monocultures, both mangrove and marsh vegetation typically increase in height in response to fertilization [22, 41]. However, in mixed mangrove-marsh assemblages, we had hypothesized that marsh plants would grow well in fertilized plots and subsequently suppress mangrove growth, based on previous work [26]. Our results did not follow our hypotheses, as individuals of both species were significantly taller in fertilized plots relative to controls in all years except 2010. *Spartina* maximum height (the only marsh parameter that significantly responded to the nutrient enrichment treatment) increased with fertilization, which is a common outcome in other *Spartina* enrichment studies [22, 42]. Nevertheless, despite that increase, average *Spartina* maximum height was shorter than *Avicennia* each year (except the first) within fertilized plots. This difference increased each year and after four growing seasons, *Avicennia* maximum height was 28% taller than *Spartina* maximum height in fertilized plots; control mangroves were only 13% taller than control *Spartina*. Mangroves are likely able to outcompete salt marsh vegetation for light because of their taller, wider canopies [43]. The taller mangrove maximum height and the reduction in *Spartina* density suggests similar competitive interactions were occurring within our fertilized plots.

Leaf nutrient content data are often used as a proxy for a fertilization response. Throughout four growing seasons of continuous enrichment, *Spartina* leaf nutrient content was unchanged, whereas *Avicennia* leaf nutrient metrics, particularly those containing nitrogen, varied between treatment plots. The positive fertilization responses in this study’s *Avicennia* leaves are similar to other mangrove-focused nutrient addition studies (e.g., [19, 20]), particularly those in nitrogen limited environments [44]. However, the lack of an enrichment response in *Spartina* leaf nutrient contents contrasts with other *Spartina* fertilization studies which have reported significant increases in tissue nutrient concentrations [22, 36]. These contrasting outcomes may be linked to the species composition of the study plots: our study plots were within mixed species stands, whereas previous work focused on monotypic stands of *Spartina*. The lack of a tissue nutrient response in our study suggests that *Spartina* nitrogen uptake may be reduced when *Avicenna* is present.

### Mangrove height and implications for coastal woody encroachment

In a meta-analysis of 273 terrestrial woody encroachment case studies, shrub height was the trait most closely correlated with ecosystem change [45]. Increases in mangrove height are likely also important in explaining how nutrient enrichment influences mangrove encroachment. In terrestrial systems, when a tree surpasses a species-specific height threshold, its ability to withstand deleterious effects from disturbances, such as fire, strengthens [46]. Likewise, mangrove heights above a certain threshold can increase mangrove tree resiliency (ability to recover) from freeze damage [47]. Negative effects on mangrove seedling growth and survivability from neighboring marsh plants [23, 24] are also lessened or even reversed after mangroves exceed a certain height [25].

Mangroves in our fertilized plots not only had significantly higher maximum heights than in control plots, but also as the enrichment period progressed, maximum height and densities of taller height classes significantly increased. Nutrient addition, by accelerating a shift in mangrove height distribution, may drive mangroves past height thresholds (that reduce negative impacts from factors such as freezing temperatures and neighboring plants) more quickly than in ambient conditions. Therefore, enriched conditions could reduce mangrove growth suppression, freezing temperature diebacks, and seedling mortality within the mangrove-marsh ecotone, subsequently facilitating mangrove stand growth and accelerating coastal woody encroachment.

### Positive feedback

Terrestrial woody plant expansion into grasslands has been documented for over a century [5]. Woody encroachment in terrestrial systems is often linked to a large-scale driver (e.g., raised CO2 levels), which changes the competitive advantage in favor of the woody plant. Concurrent changes in abiotic conditions within these ecosystems, such as increased precipitation and nitrogen deposition, can act synergistically and further facilitate terrestrial woody encroachment [7]. Some local factors (e.g., grazing) can act as a positive feedback by perpetuating woody plant coverage (e.g., reduction of grass cover by grazing ungulates) [7]. Documentation of coastal woody encroachment is much more limited than in terrestrial systems, but it is likely that local factors can promote mangrove stand growth, thereby creating positive feedbacks in coastal habitats as well [6].

**Fig 5:**
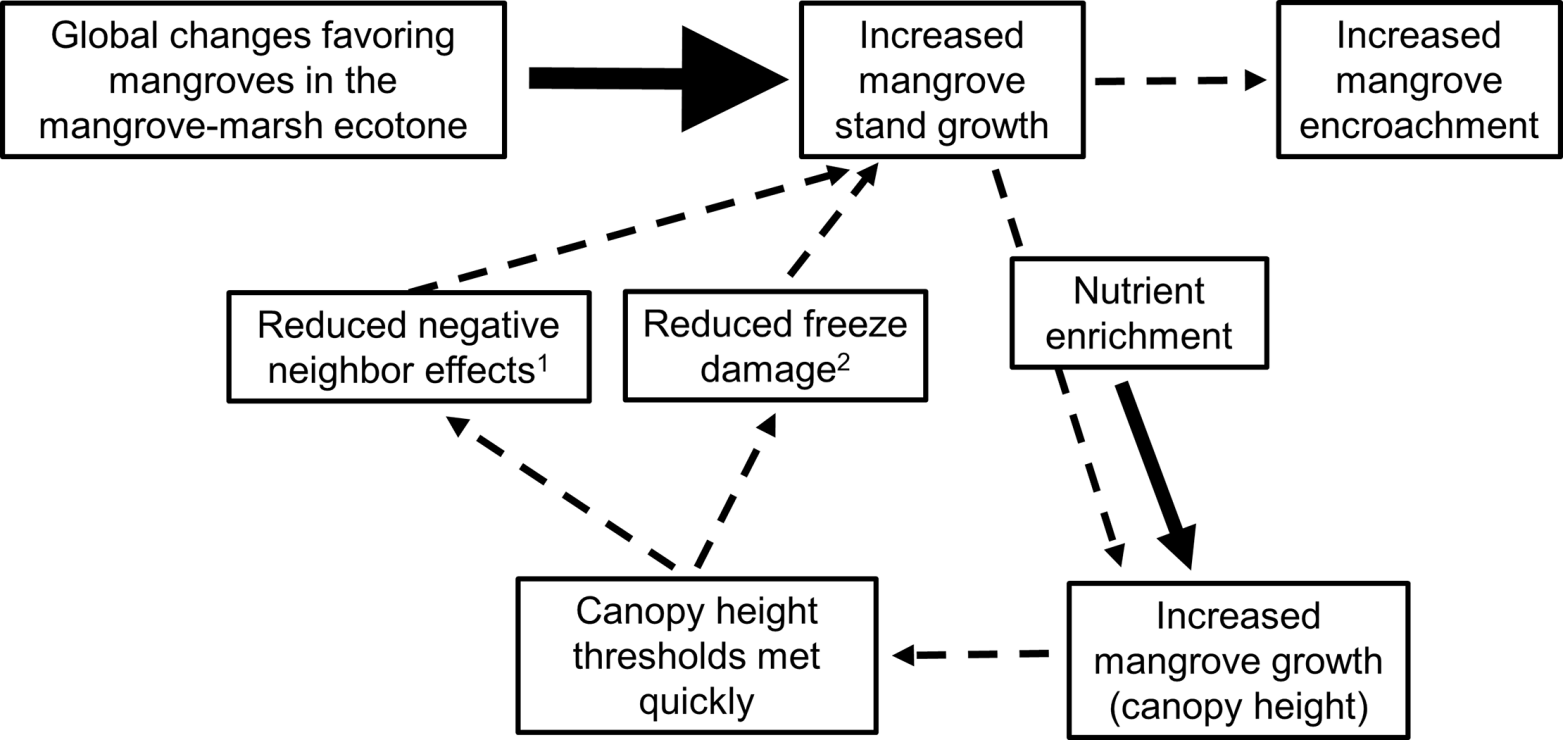
A conceptual diagram of a proposed positive feedback loop for mangrove encroachment in enriched conditions. Global changes (large bold arrow) are driving mangrove stand growth and subsequent encroachment into marshes, and this expansion may be facilitated by high nutrient conditions. Fertilization (small bold arrow) increases mangrove height, expediting mangrove canopies exceedance of species-specific growth-limiting height thresholds, such as reduced negative neighbor effects^1^ and increased resiliency to freeze damage^2^. This in turn increases mangrove stand growth and expansion. (^1^ Guo et al. 2013; ^2^ Osland et al. 2015).

Coastal woody encroachment is being driven by exogenous factors such as rising sea level and reductions in the frequency of lethal freezing events, but local factors may further influence this regime shift by accelerating mangrove growth within the mangrove-marsh ecotone. Based on our fertilization experiment, increased nutrient resource availability is likely an endogenous factor that may perpetuate mangrove stand expansion by promoting stand height to surpass thresholds related to growth suppression factors. We propose the following pathway to describe nutrient enrichment effects on mangrove encroachment dynamics: 1) global changes promote mangrove growth and stand expansion (subsequently leading to encroachment into salt marsh dominated areas); 2) nutrient enrichment stimulates mangrove growth and increases canopy height more quickly than in ambient conditions; 3) mangrove growth-limiting height thresholds (e.g., negative interactions with neighboring marsh plants and the ability to recover from freezing events) are surpassed at a faster rate; 4) reduced mangrove growth constraints promote mangrove stand survival, growth, and expansion (Fig 5).

This proposed positive feedback pathway is based on the dominant mangrove (*Avicennia*) and salt marsh grass (*Spartina*) species of the Northern Gulf of Mexico, but species-specific dynamics of mangrove encroachment vary around the world [13]. Other mangrove and marsh species may interact differently with each other and to fertilization. Additionally, mangrove encroachment within the Northern Gulf of Mexico tends to be into *Spartina* stands, which is a low elevation marsh plant. However, in other regions of the world, mangroves can encroach into salt marshes within higher elevations [48], where other endogenous factors such as soil salinity and inundation may alter nutrient responses in mangrove (e.g., [49]) and marsh (e.g., [50]) plants. Although susceptibly to freezing conditions and negative neighboring effects occur across mangrove-marsh ecotones, we recommend that this proposed feedback pathway be tested in other mangrove-marsh ecotone regions, and along a range of elevation and salinity regimes to verify nutrient enrichment favors mangrove stand expansion by alleviating mangrove growth suppression. Further, how local factors (e.g., nutrient enrichment) may act synergistically with large-scale drivers (e.g., reduced freezing temperatures and sea level rise) should be specifically tested in future enrichment work within the mangrove-marsh ecotone.

At the crux of this proposed pathway is the tenet that nutrients are augmenting mangrove height and accelerating the rate a stand will surpass various height-related growth suppression thresholds. The degree of this response and the absolute height threshold may vary by species and environmental factors. Although the current study focused on a specific set of species interactions in a Texas mangrove-marsh stand, the proposed feedback pathway and the presence of a limiting height threshold closely parallels patterns documented in terrestrial woody encroachment scenarios [7, 46]. Therefore, the conceptual framework of our positive feedback can likely be applied to mangrove encroachment in other regions, and highlights the importance of identifying regionally appropriate and species-specific height thresholds. Further, we demonstrate that nutrient enrichment may enable mangroves to surpass these limiting height thresholds at a faster rate. Therefore, coastal areas in the early stages of mangrove encroachment may be more likely to transition from marsh to mangrove dominated if in an area of higher nutrient runoff. This proposed positive feedback pathway within the mangrove-marsh ecotone can be useful in understanding the role of synergistic abiotic drivers of mangrove encroachment. A regime shift from marsh to mangrove may have large impacts on coastal ecosystem functions, with some alterations occurring rapidly [34, 51]. Therefore, it is of paramount importance to understand how local factors interact with large-scale drivers to influence this plant community shift.

## Conclusions

In this study, fertilization accelerated mangrove encroachment in a naturally co-occurring stand of *Avicennia* and *Spartina*, in a Northern Gulf of Mexico coastal area currently experiencing mangrove stand expansion [12, 32]. This outcome contrasts with previous work that documented nutrient-augmented *Spartina* growth and mangrove growth suppression when grown together [23, 26]. The positive response to fertilization in our study manifested mostly as changes in mangrove height distribution and indicates that nutrient enrichment enables smaller mangroves to grow taller, faster, and overcome growth suppression by *Spartina*. In many woody encroachment scenarios, various abiotic factors can perpetuate woody vegetation establishment and expansion. Large-scale global changes, such as sea level rise and increasing winter temperatures, are often invoked as the main driver of mangrove encroachment [13, 52], but additional nutrient resources may serve as a positive feedback for mangrove stand expansion by altering mangrove height distribution. Increases in mangrove canopy height, driven by nutrient enrichment, would allow mangroves to surpass growth-limiting height thresholds (e.g., freeze resilience and negative neighbor effects) at a faster rate and promote accelerated stand growth and expansion. Nutrient enrichment may augment mangrove encroachment, suggesting that coastal areas with higher nutrient input may be more likely to undergo this vegetation shift. Transitions within ecotones, such as woody encroachment into grass-dominated habitats, are sensitive to global changes [2]; this is particularly true in dynamic coastal systems. Therefore, it is important to identify and understand additive effects of abiotic drivers in order to better predict regime shifts under various global change scenarios.

## Supporting information

S1 Table PermANOVA results determining treatment and sampling year differences for live leaf nutrient contents. Results are from separate permANOVA to determine differences in *Avicennia germinans* (black mangrove; top portion) and *Spartina alterniflora* (smooth cordgrass; bottom portion) live leaf total carbon (% C), nitrogen (% N), phosphorus (% P), carbon to nitrogen (C:N), carbon to phosphorus (C:P), and nitrogen to phosphorus (N:P) between treatments (control and fertilized) and sampling year (2010–2013). A three-way mixed permANOVA model was utilized: treatment (2 levels) x year (4 levels) x block (11 levels). Perm p values obtained from 9999 unique permutations of the data. * Indicates significance at perm p < 0.05.
(PDF)

S2 Table Average live leaf nutrient content values. Total percent carbon (% C), nitrogen (% N), phosphorus (% P), carbon to nitrogen (C:N), carbon to phosphorus (C:P), and nitrogen to phosphorus (N:P) of live *Avicennia germinans* (black mangrove; top portion) and *Spartina alterniflora* (smooth cordgrass; bottom portion) leaves in treatment (control and fertilized) plots within each sampling year (2010–2013). n = 11 for *Avicennia* in 2010–2012 and 6 in 2013; n = 11 for *Spartina* in 2010–2011 and 9 in 2012–2013. Data are mean values (standard error).
(PDF)

S3 Table PermANOVA pairwise results comparing live leaf nutrient content values between treatments. Results are from separate pairwise permANOVA to determine treatment (control and fertilized) differences in *Avicennia germinans* (black mangrove; top portion) and *Spartina alterniflora* (smooth cordgrass; bottom portion) live leaf total carbon (% C), nitrogen (% N), phosphorus (% P), carbon to nitrogen (C:N), carbon to phosphorus (C:P), and nitrogen to phosphorus (N:P) within each sampling year (2010–2013). A three-way mixed permANOVA model was utilized: treatment (2 levels) x year (4 levels) x block (11 levels). Significance was determined for treatment within each sampling year using a pairwise test (treatment x year). Perm p values obtained from 9999 unique permutations of the data. * Indicates significance at perm p < 0.05.
(PDF)

## References

[1] Walther GR, Post E, Convey P, Menzel A, Parmesan C, Beebee TJC, et al Ecological responses to recent climate change. Nature. 2002;416(6879):389–95. doi: 10.1038/416389a PubMed PMID: WOS:000174607800036. 11919621 10.1038/416389a

[2] Risser PG. The status of the science examining ecotones. Bioscience. 1995;45(5):318–25. doi: 10.2307/1312492 PubMed PMID: WOS:A1995QU43900010.

[3] Folke C, Carpenter S, Walker B, Scheffer M, Elmqvist T, Gunderson L, et al Regime shifts, resilience, and biodiversity in ecosystem management. Annu Rev Ecol Evol Syst. 2004;35:557–81. doi: 10.1146/annurev.ecolsys.35.021103.105711 PubMed PMID: ISI:000226244100020.

[4] Scholes RJ, Archer SR. Tree-grass interactions in savannas. Annu Rev Ecol Syst. 1997;28:517–44. doi: 10.1146/annurev.ecolsys.28.1.517 PubMed PMID: WOS:000070961400020.

[5] Archer S, Schimel DS, Holland EA. Mechanisms of shrubland expansion: Land use, climate or CO_2_? Clim Change. 1995;29(1):91–9. doi: 10.1007/Bf01091640 PubMed PMID: WOS:A1995QF13100004.

[6] Saintilan N, Rogers K. Woody plant encroachment of grasslands: a comparison of terrestrial and wetland settings. New Phytol. 2015;205(3):1062–70. doi: 10.1111/Nph.13147 PubMed PMID: WOS:000348730600018. 25729806 10.1111/nph.13147

[7] D'Odorico P, Okin GS, Bestelmeyer BT. A synthetic review of feedbacks and drivers of shrub encroachment in arid grasslands. Ecohydrology. 2012;5(5):520–30. doi: 10.1002/eco.259 PubMed PMID: WOS:000309841400003.

[8] Briggs JM, Knapp AK, Blair JM, Heisler JL, Hoch GA, Lett MS, et al An ecosystem in transition: causes and consequences of the conversion of mesic grassland to shrubland. Bioscience. 2005;55(3):243–54. doi: 10.1641/0006-3568(2005)055[0243:Aeitca]2.0.Co;2 PubMed PMID: WOS:000227574400009.

[9] Van Auken OW. Causes and consequences of woody plant encroachment into western North American grasslands. J Environ Manage. 2009;90(10):2931–42. doi: 10.1016/j.jenvman.2009.04.023 . 19501450 10.1016/j.jenvman.2009.04.023

[10] Krauss KW, Lovelock CE, McKee KL, López-Hoffman L, Ewe SM, Sousa WP. Environmental drivers in mangrove establishment and early development: a review. Aquat Bot. 2008;89(2):105–27.

[11] Osland MJ, Feher LC, Griffith KT, Cavanaugh KC, Enwright NM, Day RH, et al Climatic controls on the global distribution, abundance, and species richness of mangrove forests. Ecol Monogr. 2017;87(2):341–59.

[12] Armitage AR, Highfield WE, Brody SD, Louchouarn P. The contribution of mangrove expansion to salt marsh loss on the Texas Gulf Coast. PLoS One. 2015;10(5):e0125404 doi: 10.1371/journal.pone.0125404 ; PubMed Central PMCID: PMC4422646. 25946132 10.1371/journal.pone.0125404 PMC4422646

[13] Saintilan N, Wilson NC, Rogers K, Rajkaran A, Krauss KW. Mangrove expansion and salt marsh decline at mangrove poleward limits. Global Change Biol. 2014;20(1):147–57. doi: 10.1111/gcb.12341 PubMed PMID: WOS:000327998600014. 23907934 10.1111/gcb.12341

[14] Osland MJ, Day RH, Hall CT, Brumfield MD, Dugas JL, Jones WR. Mangrove expansion and contraction at a poleward range limit: climate extremes and land‐ocean temperature gradients. Ecology. 2017;98(1):125–37. doi: 10.1002/ecy.1625 27935029 10.1002/ecy.1625

[15] Cavanaugh KC, Kellner JR, Forde AJ, Gruner DS, Parker JD, Rodriguez W, et al Poleward expansion of mangroves is a threshold response to decreased frequency of extreme cold events. Proc Natl Acad Sci U S A. 2014;111(2):723–7. doi: 10.1073/pnas.1315800111 PubMed PMID: WOS:000329614500043; PubMed Central PMCID: PMC3896164. 24379379 10.1073/pnas.1315800111 PMC3896164

[16] Nixon SW. Coastal marine eutrophication: a definition, social causes, and future concerns. Ophelia. 1995;41:199–219.

[17] Gedan KB, Silliman BR, Bertness MD. Centuries of human-driven change in salt marsh ecosystems. Ann Rev Mar Sci. 2009;1:117–41. doi: 10.1146/annurev.marine.010908.163930 PubMed PMID: ISI:000267421700006. 21141032 10.1146/annurev.marine.010908.163930

[18] Alongi DM. The impact of climate change on mangrove forests. Current Climate Change Reports. 2015;1(1):30–9.

[19] Feller IC, Lovelock CE, McKee KL. Nutrient addition differentially affects ecological processes of *Avicennia germinans* in nitrogen versus phosphorus limited mangrove ecosystems. Ecosystems. 2007;10(3):347–59. doi: 10.1007/s10021-007-9025-z PubMed PMID: ISI:000248911400001.

[20] Naidoo G. Differential effects of nitrogen and phosphorus enrichment on growth of dwarf *Avicennia marina* mangroves. Aquat Bot. 2009;90(2):184–90. doi: 10.1016/j.aquabot.2008.10.001 PubMed PMID: ISI:000261907200014.

[21] Fox L, Valiela I, Kinney EL. Vegetation cover and elevation in long-term experimental nutrient-enrichment plots in Great Sippewissett salt marsh, Cape Cod, Massachusetts: Implications for eutrophication and sea level rise. Estuaries Coasts. 2012;35(2):445–58. doi: 10.1007/s12237-012-9479-x PubMed PMID: WOS:000300771900007.

[22] Pennings SC, Stanton LE, Brewer JS. Nutrient effects on the composition of salt marsh plant communities along the Southern Atlantic and Gulf Coasts of the United States. Estuaries. 2002;25(6):1164–73. doi: 10.2307/1353160 PubMed PMID: WOS:000180611200010.

[23] Simpson LT, Feller IC, Chapman SK. Effects of competition and nutrient enrichment on *Avicennia germinans* in the salt marsh-mangrove ecotone. Aquat Bot. 2013;104:55–9. doi: 10.1016/j.aquabot.2012.09.006 PubMed PMID: WOS:000313478700007.

[24] Patterson CS, Mendelssohn IA, Swenson EM. Growth and survival of *Avicennia germinans* seedlings in a mangal salt-marsh community in Louisiana, USA. J Coast Res. 1993;9(3):801–10. PubMed PMID: ISI:A1993LK50300014.

[25] Guo H, Zhang Y, Lan Z, Pennings SC. Biotic interactions mediate the expansion of black mangrove (*Avicennia germinans*) into salt marshes under climate change. Global Change Biol. 2013;19(9):2765–74. doi: 10.1111/gcb.12221 . 23580161 10.1111/gcb.12221

[26] McKee KL, Rooth JE. Where temperate meets tropical: multi-factorial effects of elevated CO_2_, nitrogen enrichment, and competition on a mangrove-salt marsh community. Global Change Biol. 2008;14(5):971–84. doi: 10.1111/j.1365-2486.2008.01547.x PubMed PMID: ISI:000255463600003.

[27] Perry CL, Mendelssohn IA. Ecosystem effects of expanding populations of *Avicennia germinans* in a Louisiana salt marsh. Wetlands. 2009;29(1):396–406. PubMed PMID: WOS:000265294500039.

[28] Sherrod CL, McMillan C. The distributional history and ecology of mangrove vegetation along the northern Gulf of Mexico coastal region. Contributions in Marine Science. 1985;28:129–40. PubMed PMID: WOS:A1985C346500010.

[29] Sherrod CL, McMillan C. Black mangrove, *Avicennia germinans*, in Texas: Past and present distribution Contributions in Marine Science. 1981;24(9):115–31. PubMed PMID: WOS:A1981MX00700009.

[30] Markley JL, McMillan C, Thompson Jr. GA. Latitudinal differentiation in response to chilling temperatures among populations of three mangroves, *Avicennia germinans*, *Laguncularia racemosa*, and *Rhizophora mangle*, from the western tropical Atlantic and Pacific Panama. Can J Bot/Rev Can Bot. 1982;60(12):2704–15. doi: 10.1139/b82-330

[31] Montagna PA, Brenner J, Gibeaut J, Morehead S. Coastal impacts In: Schmandt J, North GR, Clarkson J, editors. The Impact of Global Warming on Texas. Austin, TX: University of Texas Press; 2011.

[32] Giri C, Long J. Is the Geographic Range of Mangrove Forests in the Conterminous United States Really Expanding? Sensors. 2016;16(12):2010. 10.3390/s16122010 PMC5190991 27916810

[33] Comeaux RS, Allison MA, Bianchi TS. Mangrove expansion in the Gulf of Mexico with climate change: Implications for wetland health and resistance to rising sea levels. Estuar Coast Shelf Sci. 2012;96:81–95. doi: 10.1016/j.ecss.2011.10.003 PubMed PMID: WOS:000300484500009.

[34] Guo H, Weaver C, Charles S, Whitt A, Dastidar S, D'Odorico P, et al Coastal regime shifts: rapid responses of coastal wetlands to changes in mangrove cover. Ecology. 2017;98(3):762–72. doi: 10.1002/ecy.1698 . 27984665 10.1002/ecy.1698

[35] Kirby CJ, Gosselink JG. Primary production in a Louisiana Gulf Coast *Spartina alterniflora* marsh. Ecology. 1976;57(5):1052–9. doi: 10.2307/1941070 PubMed PMID: WOS:A1976CN46200020.

[36] Darby FA, Turner RE. Below- and aboveground biomass of *Spartina alterniflora*: Response to nutrient addition in a Louisiana salt marsh. Estuaries Coasts. 2008;31(2):326–34. doi: 10.1007/s12237-008-9037-8 PubMed PMID: ISI:000253696500008.

[37] Fourqurean JW, Zieman JC, Powell GVN. Phosphorus limitation of primary production in Florida Bay: Evidence from C:N:P ratios of the dominant seagrass *Thalassia testudinum*. Limnol Oceanogr. 1992;37(1):162–71. PubMed PMID: ISI:A1992HV29500016.

[38] Anderson MJ. A new method for non-parametric multivariate analysis of variance. Austral Ecol. 2001;26(1):32–46. doi: 10.1111/j.1442-9993.2001.01070.pp.x PubMed PMID: WOS:000167002000004.

[39] Anderson M, Gorley R, Clarke K. PERMANOVA+ for PRIMER: guide to software and statistical methods. PRIMER-E: Plymouth, UK 2008.

[40] Nielsen-Gammon JW. The 2011 Texas drought. Texas Water Journal. 2012;3(1):59–95.

[41] Feller IC. Effects of Nutrient Enrichment on Growth and Herbivory of Dwarf Red Mangrove (*Rhizophora Mangle*). Ecol Monogr. 1995;65(4):477–505. doi: 10.2307/2963499

[42] Buresh RJ, DeLaune RD, Patrick WH. Nitrogen and phosphorus distribution and utilization by *Spartina alterniflora* in a Louisiana Gulf Coast marsh. Estuaries. 1980;3(2):111–21. doi: 10.2307/1351555 PubMed PMID: WOS:A1980JX10600006.

[43] Stevens PW, Fox SL, Montague CL. The interplay between mangroves and saltmarshes at the transition between temperate and subtropical climate in Florida. Wetlands Ecol Manag. 2006;14(5):435–44.

[44] Feller IC, McKee KL, Whigham DF, O'Neill JP. Nitrogen vs. phosphorus limitation across an ecotonal gradient in a mangrove forest. Biogeochemistry. 2003;62(2):145–75. PubMed PMID: ISI:000179363400002.

[45] Eldridge DJ, Bowker MA, Maestre FT, Roger E, Reynolds JF, Whitford WG. Impacts of shrub encroachment on ecosystem structure and functioning: towards a global synthesis. Ecol Lett. 2011;14(7):709–22. doi: 10.1111/j.1461-0248.2011.01630.x ; PubMed Central PMCID: PMC3563963. 21592276 10.1111/j.1461-0248.2011.01630.x PMC3563963

[46] Bond WJ. What limits trees in C₄ grasslands and savannas? Annu Rev Ecol Evol Syst. 2008:641–59.

[47] Osland MJ, Day RH, From AS, McCoy ML, McLeod JL, Kelleway JJ. Life stage influences the resistance and resilience of black mangrove forests to winter climate extremes. Ecosphere. 2015;6(9):1–15. doi: 10.1890/Es15-00042.1 PubMed PMID: WOS:000362121600017.

[48] Rogers K, Saintilan N, Heijnis H. Mangrove encroachment of salt marsh in Western Port Bay, Victoria: The role of sedimentation, subsidence, and sea level rise. Estuaries. 2005;28(4):551–9. doi: 10.1007/Bf02696066 PubMed PMID: WOS:000231964400007.

[49] Krauss KW, McKee KL, Hester MW. Water use characteristics of black mangrove (*Avicennia germinans*) communities along an ecotone with marsh at a northern geographical limit. Ecohydrology. 2014;7(2):354–65. doi: 10.1002/eco.1353

[50] MacTavish RM, Cohen RA. Water column ammonium concentration and salinity influence nitrogen uptake and growth of Spartina alterniflora. Journal of Experimental Marine Biology and Ecology. 2017;488:52–9.

[51] Kelleway JJ, Cavanaugh K, Rogers K, Feller IC, Ens E, Doughty C, et al Review of the ecosystem service implications of mangrove encroachment into salt marshes. Global Change Biol. 2017. 10.1111/gcb.13727 28544444

[52] Osland MJ, Enwright N, Day RH, Doyle TW. Winter climate change and coastal wetland foundation species: salt marshes vs. mangrove forests in the southeastern United States. Global Change Biol. 2013;19(5):1482–94. doi: 10.1111/gcb.12126 PubMed PMID: WOS:000317284700013. 23504931 10.1111/gcb.12126

