# Supplementary material for: Nutrient enrichment shifts mangrove height distribution: Implications for coastal woody encroachment
Source: PLoS One. 2018 Mar 1;13(3):e0193617. doi: 10.1371/journal.pone.0193617 (PMC5833200; doi:10.1371/journal.pone.0193617)
Supplement: S1 Table — Results are from separate permANOVA to determine differences in Avicennia germinans (black mangrove; top portion) and Spartina alterniflora (smooth cordgrass; bottom portion) live leaf total carbon (% C), nitrogen (% N), phosphorus (% P), carbon to nitrogen (C:N), carbon to phosphorus (C:P), and nitrogen to phosphorus (N:P) between treatments (control and fertilized) and sampling year (2010–2013). A three-way mixed permANOVA model was utilized: treatment (2 levels) x year (4 levels) x block (11 levels). Perm p values obtained from 9999 unique permutations of the data. * Indicates significance at perm p < 0.05. (PDF) [file pone.0193617.s001.pdf]

S1 Table. PermANOVA results determining treatment and sampling year differences for live leaf nutrient contents.

Avicennia germinans (black mangrove)												
	% C		% N		% P		C:N		C:P		N:P	
	Pseudo F	Perm p	Pseudo F	Perm p	Pseudo F	Perm p	Pseudo F	Perm p	Pseudo F	Perm p	Pseudo F	Perm p
**Treatment**	13.65	< 0.01*	42.98	< 0.01*	3.74	0.08	29.59	< 0.01*	0.21	0.66	45.57	< 0.01*
**Year**	14.64	< 0.01*	8.01	< 0.01*	17.36	< 0.01*	2.60	0.07	7.64	< 0.01*	2.35	0.10
**Block**	0.86	0.58	4.79	< 0.01*	1.98	0.08	3.95	< 0.01*	1.66	0.15	2.99	0.01*
**Treatment x year**	0.36	0.78	7.41	< 0.01*	1.50	0.24	5.50	< 0.01*	1.50	0.24	2.54	0.08
**Year x block**	1.69	0.10	0.93	0.57	0.74	0.76	0.87	0.63	0.79	0.71	0.94	0.56
**Treatment x block**	1.75	0.13	1.57	0.17	0.74	0.68	1.32	0.27	0.81	0.62	0.67	0.74
***Spartina alterniflora* (smooth cordgrass)**												
	% C		% N		% P		C:N		C:P		N:P	
	Pseudo F	Perm p	Pseudo F	Perm p	Pseudo F	Perm p	Pseudo F	Perm p	Pseudo F	Perm p	Pseudo F	Perm p
**Treatment**	0.96	0.41	3.01	0.12	1.11	0.35	4.68	0.05	0.91	0.37	4.21	0.07
**Year**	21.74	< 0.01*	6.05	< 0.01*	9.51	< 0.01*	5.96	< 0.01*	11.21	< 0.01*	11.03	< 0.01*
**Block**	1.43	0.25	2.93	0.02*	10.05	< 0.01*	3.51	0.01*	8.74	< 0.01*	10.89	< 0.01*
**Treatment x year**	1.45	0.26	0.50	0.69	0.60	0.64	1.24	0.34	0.62	0.61	0.44	0.73
**Year x block**	1.27	0.32	1.71	0.13	1.91	0.11	1.59	0.17	1.44	0.23	1.75	0.13
**Treatment x block**	0.91	0.54	1.38	0.26	1.65	0.19	1.61	0.19	0.63	0.77	1.00	0.48

Results are from separate permANOVA to determine differences in *Avicennia germinans* (black mangrove; top portion) and *Spartina alterniflora* (smooth cordgrass; bottom portion) live leaf total carbon (% C), nitrogen (% N), phosphorus (% P), carbon to nitrogen (C:N), carbon to phosphorus (C:P), and nitrogen to phosphorus (N:P) between treatments (control and fertilized) and sampling year (2010-2013). A three-way mixed permANOVA model was utilized: treatment (2 levels) x year (4 levels) x block (11 levels). Perm p values obtained from 9999 unique permutations of the data.

* Indicates significance at perm $p < 0.05$
