# Supplementary material for: Nutrient enrichment shifts mangrove height distribution: Implications for coastal woody encroachment
Source: PLoS One. 2018 Mar 1;13(3):e0193617. doi: 10.1371/journal.pone.0193617 (PMC5833200; doi:10.1371/journal.pone.0193617)
Supplement: S2 Table — Total percent carbon (% C), nitrogen (% N), phosphorus (% P), carbon to nitrogen (C:N), carbon to phosphorus (C:P), and nitrogen to phosphorus (N:P) of live Avicennia germinans (black mangrove; top portion) and Spartina alterniflora (smooth cordgrass; bottom portion) leaves in treatment (control and fertilized) plots within each sampling year (2010–2013). n = 11 for Avicennia in 2010–2012 and 6 in 2013; n = 11 for Spartina in 2010–2011 and 9 in 2012–2013. Data are mean values (standard error). (PDF) [file pone.0193617.s002.pdf]

S2 Table. Average live leaf nutrient content values.

***Avicennia germinans* (black mangrove)**								
**Year**	**2010**		**2011**		**2012**		**2013**	
Treatment	Control	Fertilized	Control	Fertilized	Control	Fertilized	Control	Fertilized
**% C**	44.44 (0.22)	45.33 (0.30)	45.28 (0.32)	46.25 (0.26)	46.53 (0.18)	47.60 (0.34)	44.38 (0.75)	45.73 (0.60)
**% N**	1.74 (0.06)	1.84 (0.09)	1.69 (0.07)	2.30 (0.07)	1.77 (0.06)	2.31 (0.09)	1.72 (0.13)	2.03 (0.10)
**% P**	0.13 (0.01)	0.13 (0.01)	0.13 (0.01)	0.14 (0.01)	0.14 (0.01)	0.16 (0.01)	0.15 (0.01)	0.15 (0.01)
**C:N**	30.27 (1.17)	29.39 (1.25)	31.86 (1.35)	23.68 (0.90)	31.04 (1.03)	24.40 (0.99)	30.26 (1.39)	26.72 (0.99)
**C:P**	897.51 (22.13)	912.62 (30.22)	887.28 (27.72)	834.69 (11.51)	836.01 (16.99)	790.58 (10.63)	796.05 (50.42)	829.12 (59.87)
**N:P**	29.92 (0.88)	31.33 (0.95)	28.20 (1.28)	35.69 (1.42)	27.29 (1.23)	32.91 (1.36)	26.32 (1.22)	31.01 (1.18)
***Spartina alterniflora* (smooth cordgrass)**								
**Year**	**2010**		**2011**		**2012**		**2013**	
Treatment	Control	Fertilized	Control	Fertilized	Control	Fertilized	Control	Fertilized
**%C**	40.79 (0.29)	41.47 (0.33)	41.32 (0.34)	40.91 (0.25)	40.20 (0.30)	40.75 (0.45)	42.85 (0.19)	43.28 (0.32)
**%N**	1.01 (0.07)	1.04 (0.06)	1.09 (0.11)	1.23 (0.05)	0.77 (0.07)	0.95 (0.12)	1.16 (0.07)	1.32 (0.08)
**%P**	0.13 (0.01)	0.11 (0.01)	0.18 (0.01)	0.19 (0.01)	0.18 (0.03)	0.19 (0.02)	0.15 (0.02)	0.17 (0.01)
**C:N**	49.99 (4.69)	47.98 (2.68)	47.76 (4.33)	39.46 (2.01)	66.51 (7.52)	55.05 (6.08)	44.16 (2.55)	38.98 (1.96)
**C:P**	854.89 (66.41)	961.73 (54.61)	604.34 (45.44)	592.31 (47.85)	709.25 (102.76)	607.77 (73.49)	775.61 (85.60)	672.00 (56.28)
**N:P**	17.83 (1.37)	20.53 (1.50)	13.99 (1.65)	15.00 (0.87)	11.95 (2.66)	11.98 (1.91)	60.93 (4.83)	78.46 (6.80)

Total percent carbon (% C), nitrogen (% N), phosphorus (% P), carbon to nitrogen (C:N), carbon to phosphorus (C:P), and nitrogen to phosphorus (N:P) of live *Avicennia germinans* (black mangrove; top portion) and *Spartina alterniflora* (smooth cordgrass; bottom portion) leaves in treatment (control and fertilized) plots within each sampling year (2010-2013). n = 11 for *Avicennia* in 2010-2012 and 6 in 2013; n = 11 for *Spartina* in 2010-2011 and 9 in 2012-2013. Data are mean values (standard error).
