# Supplementary material for: Nutrient enrichment shifts mangrove height distribution: Implications for coastal woody encroachment
Source: PLoS One. 2018 Mar 1;13(3):e0193617. doi: 10.1371/journal.pone.0193617 (PMC5833200; doi:10.1371/journal.pone.0193617)
Supplement: S3 Table — Results are from separate pairwise permANOVA to determine treatment (control and fertilized) differences in Avicennia germinans (black mangrove; top portion) and Spartina alterniflora (smooth cordgrass; bottom portion) live leaf total carbon (% C), nitrogen (% N), phosphorus (% P), carbon to nitrogen (C:N), carbon to phosphorus (C:P), and nitrogen to phosphorus (N:P) within each sampling year (2010–2013). A three-way mixed permANOVA model was utilized: treatment (2 levels) x year (4 levels) x block (11 levels). Significance was determined for treatment within each sampling year using a pairwise test (treatment x year). Perm p values obtained from 9999 unique permutations of the data. * Indicates significance at perm p < 0.05. (PDF) [file pone.0193617.s003.pdf]

S3 Table. PermANOVA pairwise results comparing live leaf nutrient content values between treatments.

Avicennia germinans (black mangrove)								
**Year**	**2010**		**2011**		**2012**		**2013**	
	t	Perm p	t	Perm p	t	Perm p	t	Perm p
**% C**	2.44	0.03*	2.58	0.03*	4.67	< 0.01*	1.26	0.27
**% N**	1.38	0.21	5.80	< 0.01*	5.98	< 0.01*	2.59	0.06
**% P**	0.24	0.81	2.15	0.06	3.42	< 0.01*	0.06	0.90
**C:N**	0.85	0.42	5.02	< 0.01*	5.28	< 0.01*	1.50	0.19
**C:P**	0.46	0.65	1.64	0.13	2.62	0.03*	0.41	0.69
**N:P**	1.09	0.31	3.95	< 0.01*	4.68	< 0.01*	3.61	0.02*

Spartina alterniflora (smooth cordgrass)								
**Year**	**2010**		**2011**		**2012**		**2013**	
	t	Perm p	t	Perm p	t	Perm p	t	Perm p
**% C**	1.89	0.08	0.95	0.37	1.03	0.35	1.24	0.28
**% N**	0.42	0.69	0.97	0.36	1.38	0.24	1.03	0.37
**% P**	1.99	0.07	0.01	0.99	0.90	0.43	0.04	0.96
**C:N**	0.43	0.69	1.64	0.15	1.34	0.25	1.22	0.27
**C:P**	1.76	0.11	0.30	0.78	0.62	0.54	0.49	0.64
**N:P**	2.03	0.07	1.02	0.34	2.06	0.10	0.32	0.76

Results are from separate pairwise permANOVA to determine treatment (control and fertilized) differences in *Avicennia germinans* (black mangrove; top portion) and *Spartina alterniflora* (smooth cordgrass; bottom portion) live leaf total carbon (% C), nitrogen (% N), phosphorus (% P), carbon to nitrogen (C:N), carbon to phosphorus (C:P), and nitrogen to phosphorus (N:P) within each sampling year (2010-2013). A three-way mixed permANOVA model was utilized: treatment (2 levels) x year (4 levels) x block (11 levels). Significance was determined for treatment within each sampling year using a pairwise test (treatment x year). Perm p values obtained from 9999 unique permutations of the data.

* Indicates significance at perm $p < 0.05$
